# Communication Between Autophagy and Insulin Action: At the Crux of Insulin Action-Insulin Resistance?

**DOI:** 10.3389/fcell.2021.708431

**Published:** 2021-07-15

**Authors:** Scott Frendo-Cumbo, Victoria L. Tokarz, Philip J. Bilan, John H. Brumell, Amira Klip

**Affiliations:** ^1^Cell Biology Program, Hospital for Sick Children, Toronto, ON, Canada; ^2^Department of Physiology, University of Toronto, Toronto, ON, Canada; ^3^Department of Molecular Genetics, University of Toronto, Toronto, ON, Canada; ^4^Institute of Medical Science, University of Toronto, Toronto, ON, Canada; ^5^SickKids Inflammatory Bowel Disease (IBD) Centre, Hospital for Sick Children, Toronto, ON, Canada; ^6^Department of Biochemistry, University of Toronto, Toronto, ON, Canada

**Keywords:** insulin resistance, autophagy, insulin action, obesity, type 2 diabetes, adipose tissue, skeletal muscle, liver

## Abstract

Insulin is a paramount anabolic hormone that promotes energy-storage in adipose tissue, skeletal muscle and liver, and these responses are significantly attenuated in insulin resistance leading to type 2 diabetes. Contrasting with insulin’s function, macroautophagy/autophagy is a physiological mechanism geared to the degradation of intracellular components for the purpose of energy production, building-block recycling or tissue remodeling. Given that both insulin action and autophagy are dynamic phenomena susceptible to the influence of nutrient availability, it is perhaps not surprising that there is significant interaction between these two major regulatory mechanisms. This review examines the crosstalk between autophagy and insulin action, with specific focus on dysregulated autophagy as a cause or consequence of insulin resistance.

## Introduction

### Autophagy as a Metabolic Process

**FIGURE 1:**
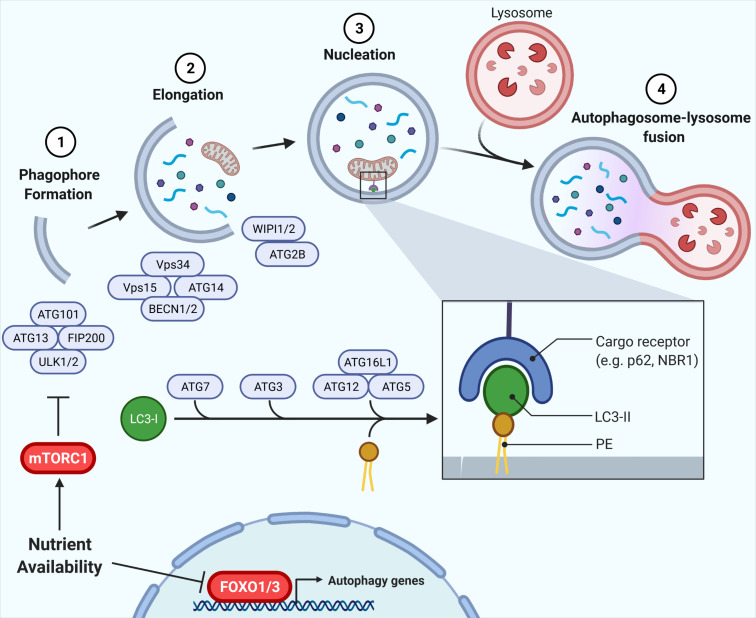
Autophagy and its regulation by nutrient availability. The autophagic process is divided into defined segments: (1) phagophore formation, (2) elongation, (3) nucleation, and (4) autophagosome-lysosome fusion. In phagophore formation, the ULK1 complex, comprising ULK1, ATG13, FIP200, and ATG101, acts as a scaffold recruiting other ATG proteins to the phagophore. The PI3K complex, including Vps34, Vps15, Beclin1/2, and ATG14L, then produces PI3P on the phagophore, promoting membrane elongation via recruitment of WIPI/ATG2. Lastly, ATG7, ATG3, and ATG12-ATG5-ATG16L1 conjugate LC3-I to phosphatidylethanolamine (PE), forming LC3-II which is vital for autophagy target recognition and autophagosome nucleation. Nutrient availability attenuates autophagy via inhibition of FOXO1 and, crucially, mTORC1 inhibition of ULK1, with the mTORC1-ULK1 and FOXO1 nodes representing critical nexus between insulin action and autophagy regulation (Shibutani and Yoshimori, 2014; Yu et al., 2018).

Macroautophagy (hereafter referred to as autophagy) is an evolutionary conserved, bulk degradation process that facilitates the deconstruction of cytosolic components, including organelles and proteins (Shibutani and Yoshimori, 2014; Yu et al., 2018). This process is initiated by the formation of a vesicular double membrane structure, termed the autophagosome, which engulfs cytosolic cargo and fuses with the lysosome, wherein internalized contents are digested (Figure 1). As such, autophagy is dependent on both the rate of flow through the vesicular pathway (autophagic flux), as well as the rate of substrate clearance by the lysosome (Loos et al., 2014). These steps are controlled by a series of autophagy-related (ATG) proteins and protein complexes such as LC3, p62, Beclin1, WIPI, ATG2, the ULK1 complex (ULK1, ATG13, FIP200, and ATG101), the PI3K complex (Vps34, Vps15, Beclin1/2, and ATG14L) and the ATG12 complex (ATG12, ATG5, and ATG16L1), illustrated in Figure 1 (Shibutani and Yoshimori, 2014; Yu et al., 2018). Studies in the past decade have highlighted the physiological importance of autophagy, identifying associations between defects in autophagy and human diseases, including diabetes and insulin resistance. The challenge is to understand how autophagy contributes to, or is impacted by disease states.

Autophagy is a highly nutrient-sensitive, catabolic process important for cellular responses to nutrient stress and, thus, it is vital in maintaining metabolic homeostasis. During starvation and caloric restriction, low nutrient availability induces autophagy to provide substrates for energy provision (Wohlgemuth et al., 2007; Russell et al., 2014). Alternatively, nutrient availability activates anabolic pathways, such as insulin signaling, that directly attenuate autophagy (Codogno and Meijer, 2005; Mammucari et al., 2007; Meijer and Codogno, 2008; Russell et al., 2014). Regulation of autophagy by nutrient plenty occurs through two primary mechanisms: (1) phosphorylation events facilitated by the kinase mTORC1, and (2) induction of gene expression via the Forkhead box O (FOXO) family of transcription factors (Mammucari et al., 2007; Zhao et al., 2007; Liu et al., 2009; Xu et al., 2011; Sanchez et al., 2012; Xiong et al., 2012; Di Malta et al., 2019; Figure 1). Downstream of mTORC1 lies the ULK1 complex, required for initiation of autophagy. mTORC1 directly phosphorylates the kinase ULK1 and its associated ATG13. In states of nutrient availability mTORC1 is activated and ULK1 and ATG13 become phosphorylated, the complex is inactive, thereby attenuating autophagy (Kim et al., 2011). During starvation, mTORC1 is inhibited, promoting dephosphorylation of ULK1 and ATG13, activating the complex to promote autophagy. In addition, ATG gene expression is highly controlled by various transcription factors that contribute to the regulation of autophagy (reviewed by Füllgrabe et al., 2016). In parallel to ULK1 inhibition, the FOXO1/3-induced expression of genes in the autophagy machinery is reduced in conditions of nutrient availability, independently of mTORC1 (Mammucari et al., 2007; Liu et al., 2009). Instead, FOXO1/3 is regulated through other kinases including AKT. Together, these phosphorylation and transcriptional responses to nutrient availability regulate autophagy induction and, in turn, autophagic flux.

### Insulin Signaling and Autophagy—A Balance of Anabolic and Catabolic Processes

Secreted postprandially in response to high nutrient availability (glucose, amino acids), insulin is critical to maintain blood glucose levels. Insulin acts by reducing hepatic glucose production in the liver and inducing disposal of dietary glucose in skeletal muscle and adipose tissue for the purpose of energy storage in the form of glycogen and triglycerides, respectively (Tokarz et al., 2018). Additionally, it is well-recognized that insulin can inhibit autophagy, through the aforementioned activation of mTORC1 leading to ULK1 phosphorylation (inhibition), and inactivation of FOXO transcription factors. In turn, ULK1 inhibits kinase activity of mTORC1 by inducing phosphorylation of raptor (Lee et al., 2007; Jung et al., 2009, 2011), creating a autoregulatory feedback loop. Recapping, the mTORC1-ULK1 nexus emerges as a key junction of insulin signaling and regulation of autophagy. Nutrient availability/insulin action activates mTORC1 leading to anabolic responses, and nutrient deprivation inhibits mTORC1 and thereby activates ULK1, leading to catabolic energy utilization. In parallel, FOXO1 integrates signaling from insulin (liberated in response to nutrient availability) by regulating autophagy gene expression.

**FIGURE 2:**
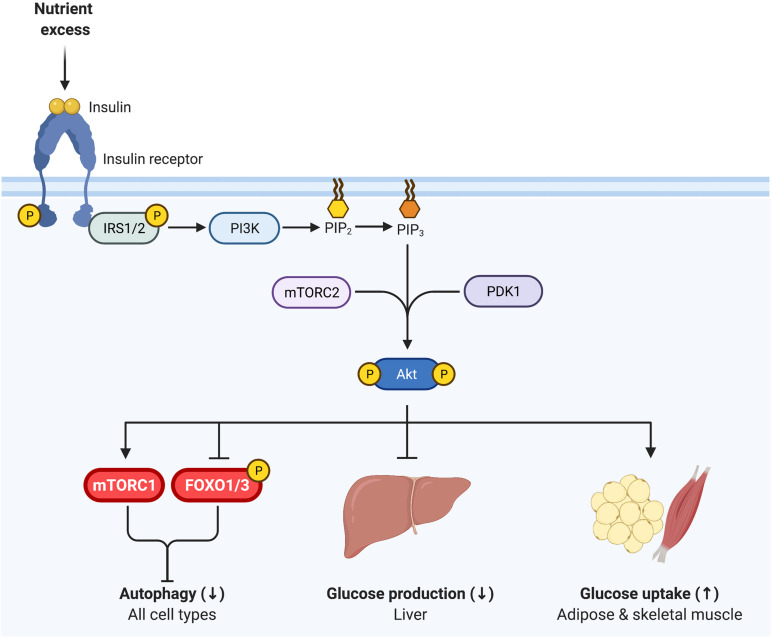
Insulin signaling regulates glycemia and inhibits autophagy. Insulin binds to the insulin receptor, inducing receptor tyrosine autophosphorylation. Insulin receptor 1 (IRS1) and IRS2 then bind to the insulin receptor and are in turn tyrosine phosphorylated. PI3K is recruited to IRS1/IRS2 and produces PI(3,4,5)P_3_ (PIP_3_) at the cell membrane, onto which AKT binds, promoting its phosphorylation by PDK1 (T308) and mTORC2 (S473). Phosphorylated AKT is active and essential for insulin inhibition of autophagy, via inhibiting FOXO1 and activating mTORC1. Thus, as in Figure 1, mTORC1 and FOXO1 are critical nexus between insulin action and autophagy regulation. AKT is also vital for insulin regulation of glycemia, representing a key signaling node in attenuating glucose production in the liver and promoting glucose uptake in skeletal muscle and adipose tissue.

The PI3K-AKT signaling pathway is a major canonical component of insulin signaling, essential for insulin inhibition of autophagy (Figure 2). Upon binding to its receptor, insulin induces receptor autophosphorylation on tyrosine residues that provide a docking site for insulin receptor substrates 1 and 2 (IRS1 and IRS2). The insulin receptor then tyrosine phosphorylates IRS1 and IRS2, inducing recruitment of PI3K and subsequent conversion of PIP_2_ to PIP_3_ at the cellular membrane. In turn, the serine-threonine kinase AKT/PKB binds PIP_3_ and is subsequently activated by mTORC2- and PDK1-mediated phosphorylation (Hemmings and Restuccia, 2012). Diverse signaling downstream of AKT induces tissue-specific outcomes of enhanced glucose disposal and diminished hepatic glucose production (Tokarz et al., 2018).

Importantly, AKT regulates both mTORC1 and FOXO1/3 activity through phosphorylation, and thereby represents a key nodule of intersection between insulin signaling and autophagy (Figure 2). AKT induces the activation of mTORC1 and inhibits FOXO1/3, consequently inhibiting autophagy (Codogno and Meijer, 2005; Mammucari et al., 2007; Meijer and Codogno, 2008). Interestingly, work in mice indicates that both deficient and enhanced autophagy lead to alterations in insulin sensitivity, as discussed in detail below. This bimodal influence suggests that the relationship between insulin signaling and autophagy is complex, and begs analysis of the contextual nature of their reciprocal cross regulation.

### Autophagy in the Development of Insulin Resistance

A poor response to insulin commonly arises with obesity and predisposes to type 2 diabetes (T2D). Insulin resistance occurs when insulin is unable to sufficiently stimulate glucose uptake in muscle and inhibit hepatic glucose production. This condition develops as a result of diminished insulin action in skeletal muscle, liver and adipose tissue, often observed as decreased phosphorylation of AKT on serine and threonine residues regulating its activity. Although the decrease in AKT activity may not be solely responsible for the ample and diverse insulin-resistant outcomes, AKT phosphorylation remains a useful index of insulin resistance. Downstream, defects in insulin signaling result in reduced activation of mTORC1 and overactivation of FOXO proteins.

In the past decade, the relationship between autophagy and insulin resistance has gained momentum. In insulin-resistant humans and mouse models of diet-induced or genetic obesity, changes in ATG gene expression and protein content have been observed in insulin target tissues, although a causal role has not been established. Here, we briefly review the changes in autophagy in insulin resistant humans and rodents, and discuss the effects of deficient autophagy on insulin signaling and whole-body insulin action in *Atg* knockout rodent models, focusing on the fundamental insulin target tissues—adipose tissue, skeletal muscle and liver.

## Adipose Tissue: The Energy Reserve

Adipocytes are fat-storing cells that regulate energy balance and glucose homeostasis in the whole organism by regulating their fat content and secreting adipokines that act on distal tissues. White adipocytes store energy as triglycerides that can be mobilized through lipolysis to release fatty acids during times of nutrient depletion of the organism, to fuel other organs. In the fed state, insulin promotes energy storage in white adipocytes by increasing their capacity to take up and store fatty acids and glucose as triglycerides in a large lipid droplet. In contrast to white adipocytes that are “energy storing,” brown adipocytes are “energy burning” (i.e., thermogenic), through their increased mitochondrial content and expression of UCP1. As their lipid is constantly turned over, it segregates into smaller lipid droplets rather than in a single large lipid droplet, and at the whole body level brown adipocytes are associated with insulin sensitization (Saely et al., 2012). Changes in white adipose tissue (WAT) autophagy impinge on the levels of “browning” of the tissue by diverting metabolism from lipid storage to lipid utilization.

The insulin-dependent uptake of fatty acids and glucose in fat cells is mediated by translocation of their respective transporters, CD36 and GLUT4, from intracellular stores to the plasma membrane (Figure 2). While only responsible for 10–15% of post-prandial glucose uptake, perturbations in adipose tissue function impair whole-body insulin sensitivity and glycemic control (as reviewed by Guilherme et al., 2008).

### Autophagy and Metabolism in Human Adipose Tissue

The relative ease to sample subcutaneous WAT in humans, compared to liver and skeletal muscle, has enabled the study of autophagy in obesity and/or diabetes in this tissue. The majority of such studies have relied on measuring protein or mRNA levels of autophagy-related proteins in whole tissue explants. However, caution must be exercised when interpreting data on the expression levels of ATGs, as expression alone is not a *bona fide* measurement of autophagic flux (reviewed by Klionsky et al., 2021). Nonetheless, those values establish a baseline of defects that a functional analysis should build upon. Estimates of autophagic flux in general involve the detection of LC3-I and LC3-II levels and abundance of LC3-II-containing particles in the cytosol, denoting autophagosome formation (Figure 1). In addition, LC3-II abundance and autophagosome accumulation are impacted by both autophagic flux and the rate of lysosomal degradation (Loos et al., 2014). Lysosomal inhibitors that act by reducing lysosomal acidification are used to measure autophagic flux, as they eliminate the influence of cargo degradation in this organelle (Shibutani and Yoshimori, 2014; Yoshii and Mizushima, 2017).

As recently reviewed by Clemente-Postigo et al. (2020), WAT from obese individuals presents elevated levels of the autophagic markers ATG5, ATG7, ATG12, LC3-II, p62, and Beclin-1, at either the mRNA and/or protein levels. This amplified expression of autophagy genes in obese WAT is likely mediated by increased activity of both FOXO and E2F family of transcription factors (Haim et al., 2015; Maixner et al., 2016). Examination of WAT explants in the presence of lysosomal inhibitors provides a more accurate measure of autophagic flux by blocking autophagosome degradation. Importantly, such studies have been performed and support that autophagic flux is in fact enhanced in WAT of obese individuals (Kovsan et al., 2011).

WAT explants contain not only adipocytes, but also fibroblasts, immune cells and vascular cells—collectively referred to as the stromal vascular fraction. Although these cells display a relative paucity of autophagy gene and protein expression compared to isolated adipocytes from WAT biopsies (Kovsan et al., 2011), measurements performed on whole explants include the joint contribution of all cells present in the tissue. Macrophages, are of particular interest given that they exhibit increased infiltration into WAT during obesity and impact insulin sensitivity and glucose homeostasis (Heilbronn and Campbell, 2008), and these cells display decreased autophagic flux in insulin-resistant mice (Liu et al., 2015a). Importantly, macrophage-specific knockout of *Atg5* or *Atg7* impairs glucose tolerance and insulin sensitivity (Liu et al., 2015a; Kang et al., 2016).

To directly assess the impact of obesity on adipocyte autophagy independent of the contributions of stromal vascular cells present in WAT biopsies, Soussi et al. (2015) isolated adipocytes from obese and control individuals. Obesity reduced LC3-II protein accumulation, indicating attenuated adipocyte autophagic flux, and that these changes were inversely correlated to fat cell size. Further, the obesity-induced reductions in adipocyte autophagy were reversible and improved after bariatric surgery (a strategy that diminishes energy intake and restores systemic insulin sensitivity). On the other hand, Öst et al. (2010) found that autophagic flux is elevated in adipocytes isolated from patients with T2D, measured by scoring phagosome content via electron microscopy and scoring LC3-II by immunofluorescence. This increased autophagic flux was associated with insulin resistance induced attenuation of mTORC1 activity (Öst et al., 2010). Of note, the latter study compared T2D subjects to a heterogenous population of control subjects consisting of lean, obese and insulin resistant subjects who did not have T2D, and were thus unable to discern differences induced by obesity from those of overt diabetes. A plausible reconciliation of the findings by Öst et al. (2010) and Soussi et al. (2015) is that adipocyte autophagy is attenuated in obesity, but as this condition progresses to T2D autophagy is conversely enhanced. In support of temporal changes in autophagy in the progression of obesity and T2D, Rodríguez et al. (2012) found that expression of *ATG7* and *BECN1* were unchanged in obese omental WAT compared to lean, while these genes exhibited increased expression in T2D WAT. However, autophagic flux, *per se*, was not assessed and therefore more work is required to confirm a functional contribution of autophagy in the diabetic phenotype. Therefore, whether adipocyte autophagic flux is increased or decreased in obesity, insulin resistance and diabetes remains controversial and may follow a biphasic pattern. Importantly, autophagic responses in various disease states are often biphasic, with measures of autophagic flux varying throughout disease progression (Schneider and Cuervo, 2014), although this has yet to be established with regard to insulin resistance and T2D. Overall, findings in whole WAT explants indicate that obesity and insulin resistance are accompanied by elevated autophagic flux and expression of autophagic markers (reviewed in Clemente-Postigo et al., 2020). Intriguingly, adipocytes isolated from tissue explants of obese individuals have lower autophagic flux than controls, unlike those from explants from type 2 diabetes (Öst et al., 2010; Soussi et al., 2015), suggesting that obesity, but not diabetes, impart adipose cell-autonomous changes in the autophagic process (Table 1).

**TABLE 1:** Impact of obesity and insulin resistance on autophagy.

	Human subjects Obesity/T2D	Rodent models HFD/*ob/ob*
Adipose tissue	**↑** PC/GE (Clemente-Postigo et al., 2020) **↑** AF (Öst et al., 2010; Kovsan et al., 2011) **↓** AF (Soussi et al., 2015)	**↑** AF/PC (Nuñez et al., 2013; Mizunoe et al., 2017) **↓** AF (Yoshizaki et al., 2012)
Skeletal muscle	**≈** PC/GE (Kruse et al., 2015) **↓** PC/GE (Møller et al., 2017)	**↑** PC (Liu et al., 2015b) **≈** PC (Turpin et al., 2009; He et al., 2012)
Liver	**≈**/**↑** PC/GE (Ezquerro et al., 2019)	**↓** AF/PC/GE (Liu et al., 2009; Yang et al., 2010) **≈** PC/GE (Ezquerro et al., 2016)

*Arrows indicate changes in autophagic flux (AF), ATG protein content (PC) and/or ATG gene expression (GE), where indicated.*

Beyond their importance as an insulin responsive energy reserve, adipocytes contribute to whole body insulin sensitivity through secretion of paracrine and autocrine acting cytokines, termed “adipokines.” In addition to the influence of alterations of autophagy on metabolism, adipocyte endocrine function is also impacted. Genetic variants in *Atg7* correlate with circulating levels of the adipokine chemerin. Mechanistically, *Atg7* knockdown in adipocytes *in vitro* reduced secretion of the chemokine chemerin (Heinitz et al., 2019). This would suggest that there is a reduction in adipocyte-mediated macrophage recruitment in conditions of deficient autophagy. Illustrating a reciprocal cellular crosstalk between cytokines and autophagy, the adipokine leptin moderately enhanced autophagosome dynamics in cultured adipocytes (Goldstein et al., 2019). This finding is of particular interest, as leptin is considered and insulin sensitizing adipokine (Amitani et al., 2013) and therefore future work should examine whether leptin mediated changes in autophagy contribute to its whole-body insulin sensitizing actions.

### Autophagy and Metabolism in Mouse Adipose Tissue

Animal models offer a degree of manipulability impossible to achieve in human studies and have been instrumental in our understanding of the relationship between autophagy and insulin resistance. Autophagy poses a critical influence on adipocyte differentiation, lipid droplet degradation (via lipophagy) and thermogenesis (Ferhat et al., 2019). Mice with adipocyte-specific congenital knockout of *Atg7*, a gene required for autophagy, display severely underdeveloped white adipocytes, with features characteristic of brown adipocytes (Singh et al., 2009b; Zhang et al., 2009). Metabolically, adipocytes from adipocyte-specific *Atg7* knockout mice display enhanced β-oxidation, leading systemic changes in free fatty acid homeostasis (Singh et al., 2009b; Zhang et al., 2009). These differentiation and metabolic changes induced by inhibition of autophagy have important implications in the context of insulin resistance.

High fat diet (HFD) feeding is a widely used strategy to drive weight gain and insulin resistance (Winzell and Ahren, 2004), and has been used to study the effects of high caloric intake on autophagy in insulin-responsive tissues. WAT from HFD-fed mice has augmented protein expression of LC3-II and p62, but not ATG5, while Beclin1 is either elevated or remains unchanged (Nuñez et al., 2013; Mizunoe et al., 2017). When autophagic flux was measured in the presence of a lysosomal inhibitors, HFD enhanced autophagic flux in WAT explants and caused accumulation of autophagosomes, presumably because clearance failed to match the rate of stimulated autophagy (Mizunoe et al., 2017). Opposite to these findings, Yoshizaki et al. (2012) reported that HFD impaired formation of LC3-II puncta in WAT. The discrepant results may be due to the length of HFD feeding, 16 weeks in Yoshizaki et al. (2012) and 30 weeks in Mizunoe et al. (2017), creating the possibility that shorter durations of HFD initially increase autophagic flux, but longer durations suppress it. Together, studies in mouse WAT mirror those of humans, as HFD increases expression of autophagic markers, but alterations in autophagic flux depend on the duration of HFD-feeding in animals or disease state (obesity *vs.* overt type 2 diabetes) in humans (Table 1).

**FIGURE 3:**
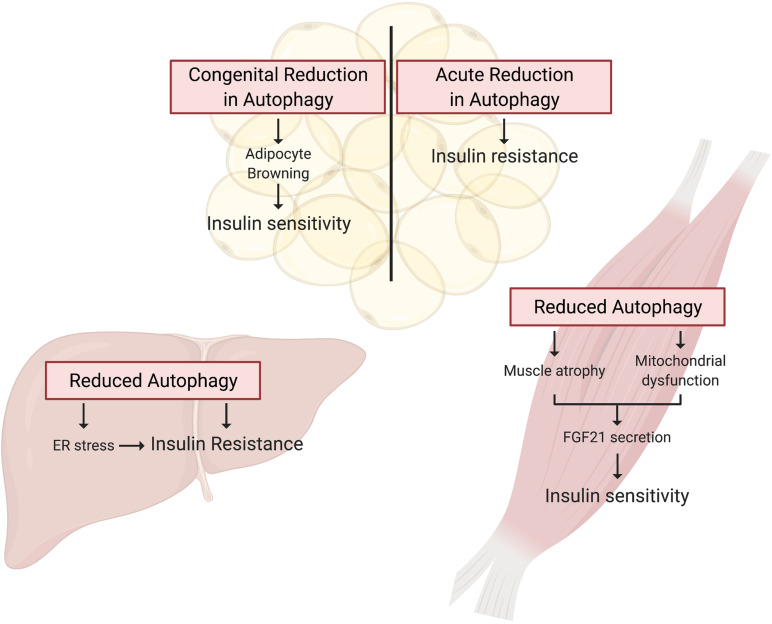
Autophagy deficient rodent models present tissue specific alterations in insulin action. Tissue specific knockout of *Atg* genes in mice has provided a wealth of knowledge guiding our understanding of the potential for deficient autophagy to induce insulin resistance. In WAT, autophagy is essential for adipocyte differentiation, and thus congenital *Atg* knockout in these cells promotes a brown adipocyte phenotype that drives whole-body insulin sensitivity. In contrast, inducible adipocyte knockout of autophagy genes in mature white adipose tissue induces insulin resistance. Autophagy is also essential for skeletal muscle maintenance and development, and thus skeletal muscle-specific *Atg* gene knockout in mice induces muscle atrophy and mitochondrial dysfunction. These muscle impairments paradoxically promote FGF21 secretion and hence whole-body insulin sensitivity. Lastly, hepatocyte-specific *Atg* knockout induces insulin resistance associated with the induction of ER stress.

While these studies indicate that overnutrition dysregulates autophagy in WAT, it is not clear whether dysregulated autophagy impairs insulin sensitivity or is a consequence of insulin resistance. To answer this question, Singh et al. (2009b) and Zhang et al. (2009) studied insulin sensitivity in mice with congenital, adipocyte-specific knockout of *Atg7*. Interestingly, loss of *Atg7* in adipocytes enhances WAT and whole-body insulin sensitivity compared to control mice, even when challenged with a HFD (Singh et al., 2009b; Zhang et al., 2009). However, in both studies, these independent groups attributed the insulin-sensitizing effects of adipocyte-specific *Atg7* knockout to “browning” of WAT brought on by the indispensable role of autophagy in adipocyte differentiation (Singh et al., 2009b; Zhang et al., 2009). Under conditions of overnutrition, these metabolically active “energy burning” brown adipocytes were protective against insulin resistance. Notably, insulin signaling to AKT was enhanced in WAT of HFD-fed adipocyte-specific *Atg7* knockout mice compared to WT controls (Singh et al., 2009b). Therefore, it appears that impaired adipocyte differentiation, not the inhibition of autophagy *per se*, is responsible for the insulin-sensitizing effects of congenital inhibition of autophagy in WAT (Figure 3).

To circumvent the detrimental effect of inhibiting autophagy on adipocyte development, and examine the effect on insulin action of a more acute reduction in autophagy Cai et al. (2018) generated mice with inducible, adipocyte-specific knockout of *Atg16l1* or *Atg3*. This system allowed them to examine the effect of inhibiting autophagy in mature adipocytes on insulin signaling. In stark contrast to the effects of congenital adipocyte autophagy inhibition, knockout of *Atg16l1* or *Atg3* in the adipose tissue of 8-week old mice with mature WAT depots caused insulin resistance (Figure 3). More specifically, autophagy inhibition in the adipocytes impaired insulin signaling to AKT not only in the WAT, but also in the liver and skeletal muscle (Cai et al., 2018). Further support for the development of insulin resistance from deficient autophagy comes from mice with adipocyte-specific depletion of p62. These mice experience increased weight gain and fat mass and have impaired glucose and insulin tolerance compared to wild-type controls (Müller et al., 2013). Complementing these findings, Yamamoto et al. (2018) reported that constitutive activation of autophagy (by whole-body expression of Beclin1^F121A^) preserved insulin signaling to AKT in WAT of HFD-fed mice by mitigating endoplasmic reticulum (ER) stress, a well described contributor to the development of insulin resistance. The mechanistic connections between autophagy and ER stress and the connection between ER stress and insulin resistance are beyond the scope of this review, but excellent reviews exist on these topics (Hotamisligil, 2010; Rashid et al., 2015; Salvadó et al., 2015). As a collective interpretation of these studies, congenital inhibition of autophagy obstructs adipogenesis and results in enhanced insulin sensitivity due to adipocyte browning, but selective inhibition of autophagy in mature adipocytes causes insulin resistance (Table 2).

**TABLE 2:** Impact of altered autophagy on insulin action.

	Congenital ATG knockout	Inducible ATG knockout	Enhanced autophagy
Adipose tissue	**↑** WB/TS (Singh et al., 2009b; Zhang et al., 2009)	**↓** WB/TS (Cai et al., 2018)	**↑** WB/TS (Yamamoto et al., 2018)
Skeletal muscle	**↑** WB (Kim et al., 2013)	NA	**↑** WB/TS (Yamamoto et al., 2018)
Liver	**↓** WB/TS (Yang et al., 2010) **↑** WB (Kim et al., 2013)	NA	**↑** WB/TS (Yang et al., 2010; Yamamoto et al., 2018)

*Arrows indicate changes in whole-body insulin action (WB) and/or tissue insulin signaling (TS), where indicated. NA, No available data.*

## Skeletal Muscle: The Energy User

Skeletal muscle primarily functions to sustain position and movement, and therefore must extract from nutrients the mechanical energy required for these processes. Despite only accounting for 40% of total body weight in humans (Frontera and Ochala, 2015), skeletal muscle is responsible for ∼80% of insulin-stimulated whole-body glucose uptake (Figure 2). Accordingly, skeletal muscle insulin sensitivity is paramount for whole body glucose homeostasis and skeletal muscle insulin resistance is a requisite precursor to the development of T2D (DeFronzo and Tripathy, 2009). Autophagy is critical for the maintenance of skeletal muscle integrity and mass (Masiero et al., 2009) and mounting evidence indicates that autophagy is also crucial for skeletal muscle energy metabolism (Neel et al., 2013).

### Autophagy and Metabolism in Human Muscle

Given the complexity expected from analyzing an integrated function such as autophagy in humans, skeletal muscle biopsies have been examined to score indices of autophagy. Perhaps not surprisingly, this has met with contrasting results across studies (Table 1). Kruse et al. (2015) found no differences between lean, obese and T2D patients in the expression of various autophagy markers (mRNA of *ULK1*, *BECN1*, *ATG5*, *ATG7*, *ATG12*, *GABARAPL1*, *p62*; proteins ATG7, BNIP3, LC3B-I, and II, p62). However, a more recent study found lower expression of autophagy-related genes (*ATG14*, *RB1CC1*/*FIP200*, *GABARAPL1*, *p62*, and *WIPI1*) and proteins (LC3-II, p62, and ATG5) in skeletal muscle biopsies from individuals with T2D, suggesting suppression of autophagy (Møller et al., 2017). Insulin infusion during hyperinsulinemic-euglycemic clamp conditions (where glucose levels were maintained at 5.5 mmol/l) lowered the content of LC3-II protein in biopsies of lean and obese humans, but not of T2D patients (Kruse et al., 2015). These findings suggest that T2D, but not obesity, disrupts insulin action on autophagosome formation. Interestingly, insulin inhibition of autophagy was restored when T2D patients were studied under isoglycemic clamp conditions (where glucose levels were maintained at the prevailing level of fasting hyperglycemia, i.e., isoglycemia) (Kruse et al., 2015). The distinct responses during euglycemia and isoglycemia in T2D patients led the authors to speculate that the failure of insulin to inhibit autophagy under euglycemic conditions represents a putative adaptation of autophagy to hyperglycemic conditions that ultimately preserves muscle mass (Kruse et al., 2015). In this context, the drop in autophagy markers in insulin resistant individuals is posited to reflect a chronic suppression of autophagy that occurs during hyperinsulinemic-hyperglycemic conditions.

### Autophagy in Mouse Muscle

Numerous studies have aimed to characterize the relationship between overnutrition, insulin resistance and autophagy (Table 1). Six weeks of HFD-feeding elevated LC3-II expression and lowered p62 protein expression, indicating that HFD induced skeletal muscle autophagy (Liu et al., 2015b). On the other hand, after 12 weeks, LC3-II levels were similar in skeletal muscle biopsies from chow- and HFD-fed mice, indicating that HFD-feeding did not alter basal autophagy (Turpin et al., 2009; He et al., 2012). A potential reconciliation of these observations would again be that varying durations of overnutrition have biphasic consequence on the regulation of autophagy, akin to that observed in adipose tissue. This scenario highlights the pressing need to resolve the temporal effects of overnutrition on skeletal muscle autophagy.

In order to directly assess whether HFD-induced insulin resistance impinges on insulin’s ability to regulate skeletal muscle autophagy, Ehrlicher et al. (2018) performed hyperinsulinemic-euglycemic clamps on HFD-fed (12 weeks) mice (or chow-fed controls) and assessed insulin suppression of autophagy by measuring the LC3-II/LC3-I ratio in skeletal muscle. Despite demonstrating that HFD-mice developed whole-body insulin resistance (measured by increased glucose infusion rate during hyperinsulinemic-euglycemic clamps), insulin suppressed autophagy in both groups to the same extent. Thus autophagy remained insulin-responsive in the muscle of obese and otherwise insulin-resistant mice. Notably, while insulin suppressed autophagy to similar levels in chow and HFD-fed mice, potential differences may have been obscured by the higher insulin concentrations needed to maintain euglycemia in the latter mice. Moreover, the aforementioned studies of autophagy in human and mouse skeletal muscle are limited in their interpretation, as autophagy flux, *per se*, cannot be measured without the use of lysosomal acidification inhibitors.

Studies in skeletal muscle-specific *Atg7* knockout mice have been instrumental in our understanding of how skeletal muscle autophagy reciprocally impinges on insulin sensitivity (Table 2). However, *Atg7* knockout results in profound muscle atrophy, emphasizing the requirement of autophagy in skeletal muscle development and maintaining skeletal muscle mass (Masiero et al., 2009; Kim et al., 2013). Thus, the delicate balance between activation and suppression of autophagy is integral to skeletal muscle development and any perturbations in this equilibrium has aggravating consequences. Despite this perturbation in skeletal muscle mass, glucose tolerance is enhanced in chow-fed, skeletal muscle-specific *Atg7* knockout mice, and these mice were protected from diet-induced insulin resistance (Kim et al., 2013). When fed HFD, these mice showed higher glucose uptake during hyperinsulinemic-euglycemic clamps compared to HFD-fed WT mice, indicating that attenuation of skeletal muscle autophagy promoted insulin sensitivity in the context of overnutrition (Kim et al., 2013). A potential explanation for the enhanced insulin sensitivity in spite of marked skeletal muscle atrophy is the concomitant upregulation of FGF21. This myokine has pleiotropic insulin-sensitizing effects (such as enhanced beta-oxidation, increased energy expenditure, and WAT browning) that collectively confer protection from diet-induced insulin resistance (Figure 3; Kim et al., 2013). Contrary to the initial hypothesis that attenuation of skeletal muscle autophagy would promote insulin resistance, this study highlights the importance of inter-organ communication in determining the metabolic outcome of tissue-specific perturbations in autophagy. Nonetheless, muscle-specific insulin action was not directly examined, and, therefore it remains unresolved whether deficient autophagy induces impaired insulin signaling in muscle.

Contrasting with the above observations, others have shown that overactivation of skeletal muscle autophagy may afford protection against diet-induced insulin resistance (Table 2). In Beclin1^F121A^ mice with constitutively active autophagy (described in the previous section), normal insulin signaling to AKT in muscle was preserved during HFD, granting protection from diet-induced insulin resistance. This protective influence was attributed to a reduction in ER stress (Yamamoto et al., 2018) which, as mentioned before, can drive insulin resistance. Summarizing, whereas the duration of the HFD may determine the consequence on skeletal muscle autophagy, the effect of insulin to attenuate autophagy is preserved in HFD-fed mice otherwise afflicted by peripheral insulin resistance.

### Muscle Cell Culture Models of Autophagy

Owing to the complex inter-organ communication networks that exist *in vivo*, as elegantly demonstrated by the above-described findings of Kim et al. (2013), cell culture models have been instrumental to discern the impact of autophagy on muscle intrinsic function. Interestingly, autophagic flux increases during C2C12 myoblast differentiation into myotubes. Resonating with the atrophy observed in *Atg7*-depleted mouse muscle described above, *Atg7* knockdown effectively blocked myoblast fusion and differentiation (McMillan and Quadrilatero, 2014). The consequence on insulin action was not explored.

Compared to animal studies, cell cultures also afford a simpler system to study the interrelation of autophagy and insulin action. In L6 skeletal muscle cells, transfection of dominant-negative ATG5K130R inhibited glucose uptake and impairs insulin signaling to IRS1 and AKT (Liu et al., 2015b). This work confirms that inhibition of autophagy is detrimental to insulin signaling in isolated skeletal muscle cells in absence of confounding contributions and signals from other tissues. We have shown that *Atg16l1* knockdown in L6 muscle cells markedly reduces the levels of the first target of the insulin receptor, IRS1. This phenotype was recapitulated in *Atg16l1-*knockout mouse embryonic fibroblasts, in which mechanistically we demonstrated proteasomal degradation of IRS1. Functionally, this was mediated by an E3 ubiquitin ligase complex composed of kelch-like 9 (KLHL9), KLHL13, and cullin 3 (CUL3) (Frendo-Cumbo et al., 2019). This complex now stands as a potential nodule connecting autophagy and insulin action beyond mTORC1.

Reciprocally to the reduction in insulin action upon alteration in autophagy, independent studies have found that insulin-resistant conditions in culture dysregulate autophagy. More specifically, high insulin/high glucose (HI/HG) treatment for 24 h provoked insulin resistance and impaired autophagy flux, both of which were relieved upon re-activation of autophagy and mitigation of ER stress by rapamycin treatment (Ahlstrom et al., 2017). Importantly, this effect was not observed in cells expressing the dominant-negative ATG5K130R, confirming that the restorative effects of autophagy activation on insulin sensitivity are autophagy-dependent. While this study did not examine whether insulin-mediated attenuation of autophagy was affected by HI/HG, treatment of L6 skeletal muscle myotubes with palmitate (a saturated fatty acid known to cause insulin resistance), prevented insulin-induced inhibition of autophagy at low doses (Ehrlicher et al., 2018).

In sum, findings in humans, mice and cell culture models identify that T2D and conditions known to induce insulin resistance (HFD feeding in mice, HI/HG and palmitate in cells) induce defects in skeletal muscle autophagy. However, whether these defects in autophagy in turn contribute to the development of insulin resistance is unclear: although muscle cell culture models of deficient autophagy display insulin resistance, mouse knockout models are complicated by inter-organ communication.

## Liver: Energy Storage and Glucose Supplier

The liver is crucial for regulating whole-body glucose metabolism through a tightly regulated balance of glucose storage and release. Under fasted conditions and post-absorptively (between meals), the liver produces glucose through *de novo* gluconeogenesis (from amino acids and other intermediates) and glycogenolysis (glycogen breakdown). However, in conditions of nutrient plenty, insulin acts on liver hepatocytes to inhibit glucose production and promote lipid storage (Figure 2). During insulin resistance, insulin is unable to inhibit hepatic glucose production, and consequently blood glucose levels rise.

Studies in the liver and isolated hepatocytes have greatly contributed to our understanding of the significance of autophagy in metabolism. Hepatocytes were one of the first mammalian cell types in which autophagy was described and are the first cells in which the effects of insulin on inhibiting autophagy were characterized. The liver was also the first tissue in which lipophagy, the specific degradation of lipid droplets by autophagy, was described (Singh et al., 2009a). Under starvation conditions, autophagy is induced principally in the liver, which over a 2-day period leads to ∼40% loss of liver protein in rodent models (Schworer et al., 1981; Mortimore et al., 1983; Ezaki et al., 2011). This upregulated hepatic autophagy is essential for the liver’s role in maintaining blood glucose levels, as it regulates both gluconeogenesis (by producing amino acids used as precursors (Ezaki et al., 2011)) and glycogen breakdown (via glycophagy). Low circulating insulin levels are thought to be the primary driver of this process (Ezaki et al., 2011), removing inhibition of autophagy in hepatocytes. Much of this glucose is then secreted, maintaining blood glucose levels and providing energy to the rest of the body.

Given the intersection between autophagy and insulin signaling in regulating liver metabolism, it is especially compelling to speculate on the potential for changes in autophagy to contribute to the development of insulin resistance in this tissue. One such study by Ezquerro et al. (2019) described increased expression of *PIK3C3* in liver of T2D patients, while *ATG5*, *BECN1*, and *ATG7* gene expression and p62 protein content were unchanged. Interestingly, the LC3-II/LC3-I increased in prediabetic patients, but was unchanged in T2D patients compared to obese insulin-sensitive subjects (Table 1). This suggests that either enhanced autophagic flux or blunted lysosomal degradation of autophagosomes may occur in the liver of prediabetic patients. As all groups compared were obese, it is unclear whether obesity *per se* induces changes in hepatic autophagy (Ezquerro et al., 2019). However, further human data examining the cause-effect relationship between deficient autophagy and insulin resistance in liver is lacking. This is likely due to the difficulty of evaluating autophagic flux in humans, as opposed to expression levels of autophagy genes, as well as to the invasive procedure required to extract liver samples from humans compared to the relative ease of sampling skeletal muscle and adipose tissue through superficial biopsies or from surgical material. Instead, rodent models form the basis of *in vivo* knowledge regarding autophagy regulation under conditions of insulin resistance.

HFD and genetic mouse models have been used to evaluate changes in autophagy in insulin resistant states (Table 1). When measured in the absence of lysosomal inhibitors, the LC3-II/LC3-I ratio and p62 content were unchanged in rats fed a HFD for 4 months (Ezquerro et al., 2016). However, autophagic flux (measured following *in vivo* treatment with chloroquine) decreased in the liver of *ob/ob* and HFD-fed mice, denoted by reduced LC3-II/LC3-I ratio, increased p62 accumulation and reduced autophagosome formation (Liu et al., 2009; Yang et al., 2010). Moreover, the expression of autophagy-related genes (*Vps34, Atg12, and Gabarapl1*) and of proteins LC3, Beclin 1, ATG5, and ATG7, was significantly lower compared to control mice, although gene expression of *Beclin1*, *Ulk2, Atg5, Sqstm1*, and *Atg7* was unaffected (Liu et al., 2009; Yang et al., 2010; Ezquerro et al., 2016). These features were associated with the development of insulin resistance and concomitant hyperinsulinemia. Causally, Liu et al. (2009) proposed that hyperinsulinemia is the main contributor to deficient autophagy, since treating mice with a PI3K inhibitor (LY294002) recovered defects in autophagic flux. Assuming that the hyperinsulinemia of *ob/ob* mice may have driven the altered autophagy, Yang et al. (2010) explored the effect of normalization of insulin levels using the pancreatic toxin streptozotocin (STZ). However, ATG7 levels were not restored, and instead the study suggested the elevated calpain 2 expression was responsible for ATG7 degradation, while autophagic flux was not measured.

To more precisely examine the consequence of the obesity-linked drop in ATG on insulin action, ATG7 was overexpressed in the liver of HFD and *ob/ob* mice. This treatment concomitantly improved insulin-stimulated phosphorylation of the hepatic insulin receptor and AKT, and reduced hepatic glucose production (Yang et al., 2010), ultimately improving skeletal muscle glucose uptake and whole-body insulin sensitivity. Mechanistically, ER stress and hepatic lipid accumulation, considered contributors to the development of hepatic insulin resistance, were curbed (Yang et al., 2010). Importantly, co-expression of dominant-negative ATG5 (ATG5K130R) that is unable to bind ATG12 and thus attenuates autophagic flux, abolished the beneficial effects of ATG7 on liver insulin signaling. These findings suggest that changes in autophagy are responsible for the fluctuations in insulin sensitivity upon changes in ATG7 expression.

Complementing this conclusion, hyperactivation of autophagy via whole-body expression of Beclin1^F121A^ improved overall insulin sensitivity in HFD-fed mice, associated with reduced hepatic ER stress (Yamamoto et al., 2018). This study therefore supports the notion that the effects of autophagy on insulin sensitivity are mediated by the level of ER stress, and specifically that deficient autophagy promotes ER stress and subsequent insulin resistance.

Additional studies explored the effects of genetic attenuation of autophagy on insulin signaling (Table 2). Adenovirus-mediated ablation of *Atg7* in liver of mice lowered insulin-stimulated phosphorylation of the insulin receptor and AKT, accompanied by whole-body insulin resistance (Figure 3; Yang et al., 2010). However, contrasting findings were observed in liver-specific *Atg7* knockout mice, which displayed protection from diet-induced obesity and insulin resistance (Kim et al., 2013), although this study did not examine insulin signaling in the liver. The observed resistance to HFD-induced obesity and insulin resistance were associated with induction of FGF21, mirroring what was observed in muscle-specific *Atg7* knockout mice, as discussed in the previous section. Interestingly, liver-specific knockout of both *Atg7* and focal adhesion kinase family kinase-interacting protein of 200 kDa (*Fip200*) reduced starvation- and HFD-induced hepatic lipid accumulation, attributed to attenuation of the *de novo* lipogenic program in the liver (Kim et al., 2013; Ma et al., 2013). Thereby, the mice were protected from HFD induced hepatic steatosis. However, this finding remains controversial as others have shown augmented hepatic lipid accumulation in these liver-specific *Atg7* knockout mice following starvation, associated with defects in lipophagy – lipid droplet specific autophagy (Singh et al., 2009a). Therefore, it remains possible that defects in autophagy have an adverse impact on *de novo* lipogenesis in conditions that promote energy storage, reducing lipid accumulation and thereby the development of insulin resistance. Conversely, under catabolic conditions defects in autophagy prevent the rapid degradation of lipid droplets and release of lipids for energy provision, leading to an increase in lipid accumulation.

## General Lessons Learned

**FIGURE 4:**
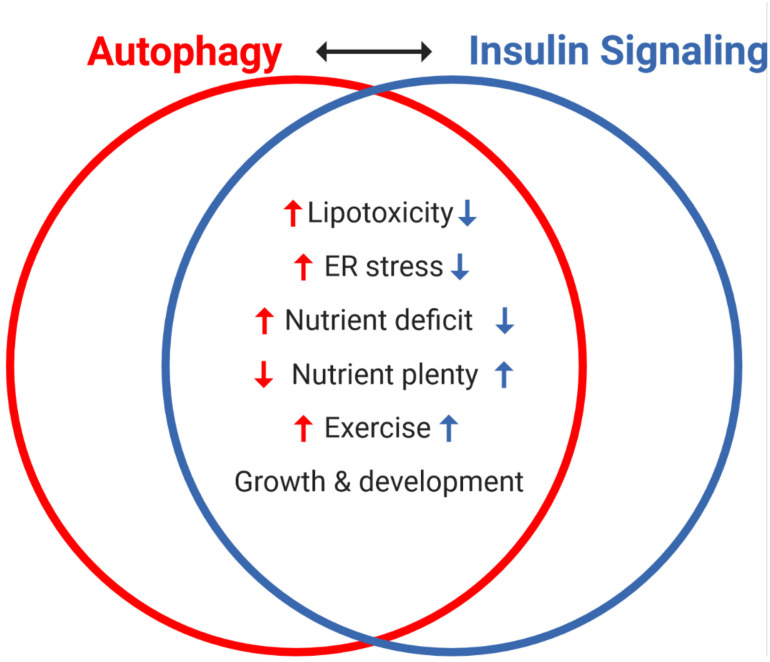
Reciprocal regulation of autophagy and insulin signaling by various interventions and cellular states. The antagonism between autophagy and insulin signaling is a powerful representation of the homeostatic balance between catabolic and anabolic processes. Notably, autophagy and insulin signaling are reciprocally impacted by various stresses: lipotoxicity and ER stress induce insulin resistance, which independently promotes autophagy. Nutrient availability is a key regulator of both processes, with acute nutrient plenty promoting insulin secretion and thereby whole-body insulin action, and conversely starvation (nutrient deficit) inducing autophagy. Interestingly, exercise (not discussed here) promotes autophagy and insulin sensitivity in skeletal muscle and both processes are important in growth and development.

Insulin signaling and autophagy are a compelling representation of the homeostatic *yin* and *yang* between anabolic and catabolic processes. The antagonism between these two pathways is exemplified by the well described inhibition of autophagy by insulin signaling, as well as the numerous pathways that reciprocally regulate these processes (Figure 4). Central to this crosstalk is the mTORC1-ULK1 connection, which can be considered as a nexus between these pathways, along with FOXO1-directed transcriptional regulation. Moving forward, the levels, location and/or frequency of mTORC1 activation, coupled to its graded engagement of ULK1, may help define the crosstalk between these two processes.

The work presented herein examined consequences of altered autophagy on insulin signaling and further highlighted the complex cross regulation of these pathways (Table 2). Many studies in insulin-target tissues implicate defects in autophagy as potential contributors to the development of insulin resistance (Table 1). Therefore, attenuated autophagy may induce negative feedback inhibition of insulin signaling, overcoming the inhibitory action of insulin on autophagy.

Important progress has been made to disclose the consequence of obesogenic and diabetic conditions on autophagy, and in turn the contribution of changes in autophagy to insulin resistance. However, our understanding of how deficient autophagy contributes to insulin resistance is incomplete and further examination is required in order to:

(a) Establish whether the autophagic process, as opposed to ATG expression alone, is in fact altered in T2D humans. To this end, Yang et al. (2010) provided the first evidence that defects in autophagic flux *per se* induces insulin resistance, rather than the loss of non-canonical functions of *Atg7*. More studies should complement examination of this involvement in the various specific-*Atg* knockout models discussed herein.
(b) Firmly establish if autophagic flux responds in a biphasic fashion during the development of insulin resistance. The studies analyzed support this possibility in aggregate, but the concept must be examined longitudinally in the same experimental system.
(c) Unravel the complete suite of molecular mechanisms through which deficient autophagy induces insulin resistance *in vivo*. In this regard, cell culture *Atg* gene-knockout models have already provided compelling evidence of how deficient autophagy induces insulin resistance (Yang et al., 2010; Liu et al., 2015b; Frendo-Cumbo et al., 2019). An important node of regulation, in addition to mTORC1-ULK1 is the control of IRS1 -life through the engagement of the ubiquitin-proteasomal machinery (Frendo-Cumbo et al., 2019). Superimposed on this direct molecular target are the indirect mechanisms of enhanced ER stress, inflammation and/or lipotoxicity, induced upon loss of autophagy and induce insulin resistance (Figure 4).

## Conclusion

Our understanding of the crosstalk between autophagy and insulin action is mostly founded on investigations in adipose, muscle and liver of animal models, as well as cell culture models of these tissues. Future studies should clarify discrepant findings and important questions remain regarding the contribution of impairments in autophagy to the development of insulin resistance and T2D. Regardless of the mechanisms, it is clear that upregulating autophagy consistently improves insulin sensitivity (Yang et al., 2010; Pyo et al., 2013; Yamamoto et al., 2018). Therefore, whether deficient autophagy is a cause or consequence of insulin resistance, or if it is in fact deficient in insulin resistance in humans, the therapeutic potential of autophagy induction to help treating insulin resistance and diabetes cannot be overlooked.

## Author Contributions

AK led the conception and design of the review. SF-C and VLT contributed to the design and wrote the first draft of the manuscript. All authors contributed to manuscript revision, read, and approved the submitted version.

## Conflict of Interest

The authors declare that the research was conducted in the absence of any commercial or financial relationships that could be construed as a potential conflict of interest.
